# Lifespan of Splints in a Sample of German Soldiers Hospitalised with Post-traumatic Stress Disorder (PTSD) in Combination with Sleep Bruxism and Painful Temporomandibular Disorder (TMD)

**DOI:** 10.3290/j.ohpd.b5569645

**Published:** 2024-07-12

**Authors:** Felix Wörner, Thomas Eger, Ursula Simon, Alexander Becker, Anne Wolowski

**Affiliations:** a Dentist, Department of Dentistry-Periodontology, Bundeswehr Central Hospital Koblenz, Koblenz, Germany. Conceptualisation, formal analysis, investigation, methodology, writing, and review.; b Dentist and Department Head, Department of Dentistry-Periodontology, Bundeswehr Central Hospital Koblenz, Koblenz, Germany. Conceptualisation, investigation, methodology, writing, review, and editing.; c Dentist and Department Head, Department and Center for Mental Health and Psychiatry, Bundeswehr Central Hospital Koblenz, Koblenz, Germany. Conceptualisation, formal analysis, investigation, methodology, writing, review, and editing.; All authors read and approved the published version of the manuscript.

**Keywords:** bruxism, PTSD, splint therapy, TMD

## Abstract

**Purpose:**

This cross-sectional longitudinal observational study aimed to clarify the question of whether painful temporomandibular disorders (TMD) in psychiatrically confirmed patients hospitalised for post-traumatic stress disorder (PTSD) therapy after using splint therapy (ST) show long-term therapeutic effects in the case of functional disorders.

**Materials and Methods:**

One hundred fifty-three (153) inpatients (123 male and 20 female soldiers, age 35.8 ± 9.2 years, 26.6 ± 2.2 teeth) with confirmed PTSD (Impact of Event Scale – Revised ≥33), grade 3 to 4 chronic pain according to von Korff’s Chronic Pain Scale and the research diagnostic criteria of painful TMD (RDC-TMD) were recorded. All participants received a maxillary occlusal splint that was worn at night. Control check-ups of the therapeutic effect of the splint were conducted for up to 9 years during psychiatric follow-ups.

**Results:**

TMD pain worsened in 22 (14.4%) patients within the first 6 weeks and led to the removal of the splint. The pain intensity (PI) at BL was reported to be a mean of VAS 7.7 ± 1.1. Six weeks after ST (n = 131), the average PI was recorded as VAS 2.6 ± 1.3. Based on the last examination date of all subjects, the average PI was recorded as 0.7 ± 0.9. Seventy-two (72) patients used a second stabilisation splint in the maxilla after 14.4 ± 15.7 months, and 38 patients used between 3 and 8 splints during their psychiatric and dental treatment time (33.7 ± 29.8 months).

**Conclusion:**

The presented data shows that therapeutic pain reduction remained valid in the long term despite continued PTSD. The lifespan of a splint seems to be dependent on individual factors. Long-term splint therapy appears to be accepted by the majority of patients with PTSD and painful TMD.

Stress can sometimes act as a direct aetiological agent in disease. PTSD, for example, results from a well-defined relationship between a stressful life event and the onset of a clinical syndrome. It is a pathological response to stress that can occur after a person experiences or witnesses a traumatic event, during which the individual is in a permanent state of increased tension. PTSD is a disorder that may develop following exposure to an extraordinarily threatening or horrific event or series of events.^41^ It is characterised by the following: 1) re-experiencing the traumatic event or events in the present in the form of vivid intrusive memories, flashbacks, or nightmares. These are typically accompanied by strong or overwhelming emotions, particularly fear or horror, and strong physical sensations; 2) avoidance of thoughts and memories of the event or events, or avoidance of activities, situations, or people reminiscent of the event or events; and 3) persistent perceptions of heightened current threat, for example, as indicated by hypervigilance or an enhanced startle reaction to stimuli such as unexpected noises. The symptoms persist for at least several weeks and cause significant impairment in personal, family, social, educational, occupational, or other important areas of life.^6^^,^^18^^,^^41^ Epidemiologic estimates of the prevalence of the various trauma sequelae disorders vary considerably depending on the sample and timepoint studied, among other factors.^41^ PTSD can cause a person to feel anxious long after the event has ended.^41^

In many cases, the first psychiatric evaluation of affected patients does not occur until years after the trauma.^6^^,^^18^^,^^41^ Dental problems in soldiers with PTSD include blood and injury phobia, dental anxiety, grinding the teeth, restricted mouth opening, painful temporomandibular joint (TMJ) clicking, dentin hypersensitivity, and marked tobacco use.^6^^,^^13^^,^^44^

The condition is accompanied by an imbalance of neurotransmitters, which may be directly or indirectly connected with the occurrence and course of TMD.^4^ The noradrenergic system disturbances, hypothalamic–pituitary–adrenal axis alterations, and disturbance in the level of serotonin could influence TMJ function by inducing muscular hyperfunction and altered pain perception.^43^

The exact nature of the relationship between psychological disorders and TMD is not fully understood; however, a strong relationship is to be assumed. Individuals with psychological disorders may be more likely to develop TMD because of the increased stress and anxiety they experience.^3^ This stress can lead to grinding the teeth (bruxism) and other habits that can damage the jaw and lead to TMD. Kindler et al^21^ estimated the association between signs of temporomandibular disorders and symptoms of post-traumatic stress disorder (PTSD) in a representative sample from the general population of north-eastern Germany. Subjects having clinical PTSD (n = 62) had a 2.56-fold increase in joint pain and a 3.86-fold increase in muscle pain compared to subjects having no clinical PTSD.

PTSD is associated with painful TMD and may be part of the aetiology of awake as well as sleep bruxism. Investigating the associations between PTSD symptoms on the one hand and painful TMD/bruxism on the other can help tailor treatment to the needs of this patient group.^22^

Bruxism is a multifaceted phenomenon associated with several factors mediated by the central nervous system. According to an updated international consensus in 2018, bruxism is a repetitive masticatory muscle activity that is not necessarily a disorder in healthy individuals.^24^ The prevalence of bruxism in the general population is approximately 25%.^27^ The effects of bruxism and associated factors on stomatognathic structures were considerably heterogeneous and inconsistent. Factors consistently associated with bruxism were the use of alcohol, caffeine, tobacco, some psychotropic medications, oesophageal acidification, and second-hand smoke; temporomandibular disorder signs and symptoms presented a plausible association.^29^ Current knowledge is mainly related to sleep bruxism (SB).

The physical and psychological effects of war are not always easy to detect, but they can be far-reaching and long-lasting. Dental treatment needs for combat- and non-combat-induced PTSD, as well as the use of different splints and exercise therapy, are not yet differentiated for the primary outcome of pain reduction and the secondary outcome of mouth opening. The frequency of splint replacement is based on the wide variation in expert opinion (0, 2, 5, and 20 years). Long-term medical data beyond 12 months does not exist but is an important driver of cost-effectiveness.^39^^,^^40^

## Aim of the Study

This cross-sectional longitudinal observational study aimed to clarify the question of whether mouth-opening restrictions in patients with PTSD and SB, after two days of exercise with self-performed massage therapy using different types of splint therapy (ST), showed long-term therapeutic effects in the case of functional disorders. The long-term impact of bruxism on TMD pain states, as well as the frequency of splint replacement, were to be determined. Patients with painful TMD not using a splint were analysed as a control group for the effect of mouth-opening and pain-reduction after exercise therapy^38^ comprising muscle-strengthening, stretching, jaw-opening, postural, and mobilisation exercises.

## MATERIALS AND METHODS

This cross-sectional observational study was conducted at the Bundeswehr Medical Service Academy (Munich, Germany) and registered in the military clinical trial register (12K1-S-80 1414). All soldiers referred for psychiatric reasons, who had been suffering from PTSD (Impact of Event Scale – Revised ≥33^1^^,^^10^) for at least 3 months, and were receiving dental treatment for bruxism and painful TMD were recruited. A trained dentist examined all patients between August 2014 and Juli 2023 at the Bundeswehr Central Hospital Koblenz, Germany. Exclusion criteria were pregnancy, known former use of narcotic substances, and acid-reflux-induced erosions. TMD pain worsened in 22 patients within the first 6 weeks and led to the removal of the splint.

Clinical dental examinations for decayed missing and filled teeth (DMFT), full-mouth periodontal status, and functional analysis of the TMJ were performed. Self-administered questionnaires were used to determine smoking habits and medical, dental, and social history. The anamnesis for bruxism and differentiation between awake and sleep bruxism was taken in a personal interview.^36^

The presence of dysfunctional symptoms was diagnosed clinically by muscle palpation according to the guidelines on RDC-TMD. Only one trained dentist performed this exam to minimise variance. A modified Tooth Wear Index (mTWI)^6^^,^^50^ on casts was used to quantify attrition. Here, a mean mTWI was calculated for each patient from the attritions of all measurable teeth not restored with a crown. Comparable measurements for crown length and width on 17- to 21-year-old males by Björndal et al^5^ were used for calibration. After oral hygiene instruction and plaque disclosure, all participants in the study received a professional cleaning or sufficient non-surgical periodontal therapy. In cases with functional symptoms in relation to pain and bruxism (131 PTSD patients), conservative treatment of occlusal dysfunctions with a maxillary open-bite aid for nocturnal use (modified acrylic interceptor) or a maxillary hard stabilisation splint (Michigan splint) was performed.^42^^,^^51^

The study’s primary outcome measurement was pain, determined via a visual analogue scale. The mean and standard deviation VAS scores served as the treatment effect, using a standardised mean difference if different scales were used (e.g., pain could be measured as pain experienced now or the worst pain experienced over the previous month). All patients fulfilled the required inclusion criteria of at least grade-3 chronic pain according to von Korff’s Chronic Pain Scale^48^ and a pain sensation of at least 6 on an initial examination VAS from 0 to 10, a myogenic restricted oral opening of less than 40 mm, which was also subjectively rated as restricted, and pain in mandibular movements experienced as impaired, as well as attrition, averaging one-third of the clinical crown height.^50^

Subjects were invited to follow-up visits after 1 week, 2 weeks, 6 weeks, 3 months, 6 months, and 12 months, and then every 6 months for up to 9 years after splint insertion and regular nocturnal wear. During these examinations, the mouth opening was rechecked after 6 weeks, and each subject filled out a VAS for PI at each follow-up appointment. The exclusion of muscle and joint diseases was based on the RDC-TMD. In cases of fracture/perforation/loss of the splint and TMJ pain-caused impairment, patients received a new open-bite aid for nocturnal use.

The statistical analysis was performed using statistical software (SPSS 24, IBM; Armonk, NY, USA). Descriptive data are presented as means ± SD or n (percentage).

The significance level was set at p < 0.05. The sample size was based on the total number of hospitalised or ambulatory patients referred by the Department of Mental Health with orofacial dysfunctional symptoms, chronic pain, and a more than 5-day period of teeth grinding for splint therapy from August 2014 to July 2023. All patients had to have been treated in the hospital or outpatient facility for more than 3 months.

### Ethics Statement

Clinical questionnaires, examinations, and multiple patient-dentist discussions in the Department for Dentistry-Periodontology of the Bundeswehr Central Hospital Koblenz were used to conduct the present investigation. The prospective study was conducted at the Bundeswehr Medical Service Academy. In full accordance with ethical principles, the guidelines of the Helsinki Declaration were followed, and the Regional Ethics Review of the State Chamber of Physicians of Rhineland-Palatinate in Germany (837.068.14/9307-F) approved the study (28 March 2014). Subjects were informed that they could leave the study at any time without consequence. All participants were military personnel. Written informed consent was obtained from all subjects involved in the study after providing referred patients with written informational material.

## RESULTS

One hundred fifty-three (153) PTSD patients were recruited (Table 1).

**Table 1:** Baseline anamnestic and clinical data in long-term PTSD therapy (n: 153)

	PTSD men (A)	PTSD women (B)
Number	123	30
Age (years)	36.0 ± 8.9	35.1 ± 10.8
Smoking percentage	63%	43%
PTSD combat-associated	85%	47%
Number of teeth	26.4 ± 2.3	27.2 ± 1.3
DMFT	10.3 ± 7.2	8.1 ± 6.5
Prophylaxis sessions in the last 2 years	0.9 ± 1.1	1.0 ± 1.1
Educational level		
≥12 years	26%	53%
10 years	43%	37%
≤9 years	31%	10%
mTWI	3.7 ± 0.8B	3.2 ± 0.8
Hospital PTSD treatment time (months)	26.4 ± 28.8	39.7 ± 34.8A
Time since last traumatic event (years)	4.7 ± 3.5	4.4 ± 3.7
Splints (n)	1.7 ± 1.4	1.9 ± 1.4
No splint therapy accepted by the patients	18	4

Results are based on two-sided tests assuming equal variances. For each significant pair, the key of the smaller category appears in the category with the larger mean. Capital letters indicate statistical significance at 0.05. Tests are adjusted for all pairwise comparisons within rows using the Bonferroni correction.

The average time between the last traumatic event in relation to PTSD and the first psychiatric presentation at the hospital was 4.7 ± 3.6 years.

Initial (BL) mouth opening was an average of 32.4 ± 8.9 mm. The pain intensity (PI) at BL was reported to have a mean score on the VAS of 7.7 ± 1.1. Six weeks after the ST (n = 131), the average mouth opening was 48.1 ± 6.4 mm, and PI was recorded 2.6 ± 1.3 on the VAS. Based on the last examination date of all subjects, the average PI was recorded as 0.7 ± 0.9.

Male and female patients with ST of more than 3 months (n = 131) did not differ statistically significantly in terms of age (36.0 ± 9.1 years), number of teeth (n: 26.6 ± 2.0), and dental care status (DMFT 9.8 ± 7.0). The extent of attrition at baseline in the male group and the PTSD treatment time in the female group were higher (Table 1).

**Fig 1:**
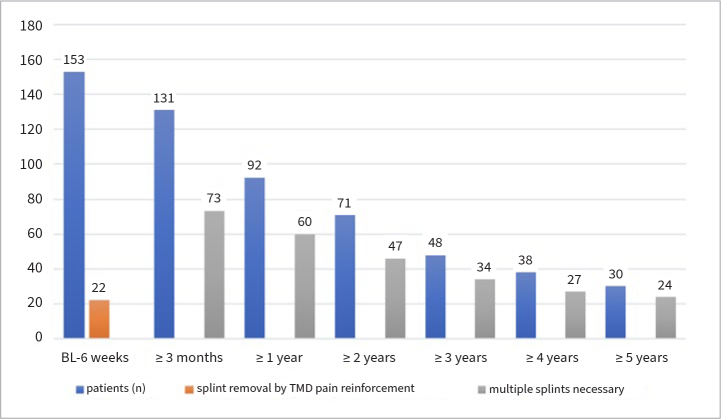
Compliance with splint therapy in PTSD patients with TMD.

TMD pain worsened in 22 (14.4 %) patients within the first week and led to the removal of the splint (Fig1). This group without splints treated themselves with self-performed massage and exercise and was separately analysed as a control group after 6 weeks (Table 2).

**Table 2:** Baseline anamnestic and clinical results after 6 weeks PTSD-therapy for patients without (control n = 22) and with splint therapy (n = 131)

	Control group		Splint therapy group	
	Non-combat PTSD (A)	Combat-induced PTSD (B)	Non-combat PTSD (C)	Combat-induced PTSD (D)
Number	5	17	30	101
Age	26.0 ± 4.3	37.8 ± 10.0A	32.9 ± 10.4	36.9 ± 8.6C
IES revised	33.0 ± 0	52.2 ± 4.3A	37.3 ± 5.3	50.9 ± 7.3C
Time since last traumatic event (years)	0	5.3 ± 4.9A	1.0 ± 0	4.9 ± 3.3 C
mTWI	2.9 ± 0.6	3.6 ± 0.6 A	3.39 ± 0.8	3.75 ± 0.8C
Mouth opening (mm) BL vs 6 weeks	37.0 ± 9.1 vs 42.0 ± 7.6	42.2 ± 11.8 vs 43.8 ± 7.9	30.8 ± 7.8 vs 46.6 ± 4.5	30.8 ± 7.4 vs 48.5 ± 6.8
Pain (VAS 0-10) BL vs 6 weeks	6.8 ± 3.0 vs 4.0 ± 1.2	7.8 ± 0.8 vs 4.5 ± 1.3	7.4 ± 1.3 vs 2.7 ± 1.7	7.8 ± 1.1 vs 2.6 ± 1.2
Splints (n) during therapy	0	0	1.9 ± 1.0	2.1 ± 1.4

Results are based on two-sided tests assuming equal variances. For each significant pair, the key of the smaller category appears in the category with the larger mean. Capital letters indicate statistical significance at 0.05. Tests are adjusted for all pairwise comparisons within rows using the Bonferroni correction.

A second splint became necessary during the observed dental treatment time in 73 patients. After 3 months, 1, 2, 3, 4, and 5 years, 131, 92, 71, 48, 38, and 30 subjects, respectively, were interviewed regarding pain intensity (PI).

**Fig 2:**
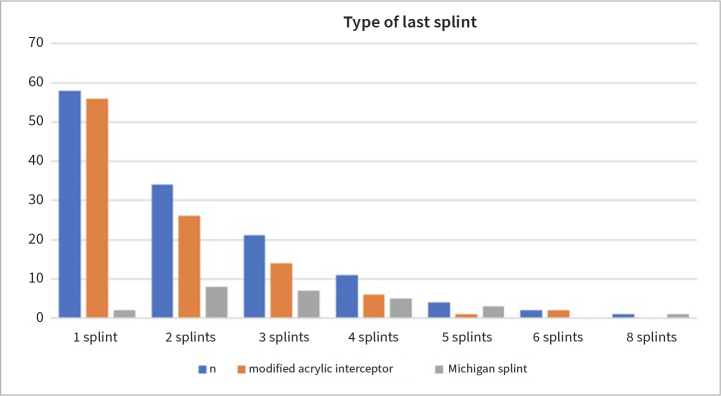
Number and type of last TMD splint used (n = 273 splints in 131 patients) within the hospitalised PTSD treatment period.

Seventy-two (72) patients used a second splint for the maxilla after 14.4 ± 15.7 months, and 38 patients received between 3 and 8 splints during their psychiatric and dental treatment (33.7 ± 29.8 months). Thirty-three (33) of 71 PTSD patients re-examined after 2 years used a second splint after 9.3 ± 7.2 months. After 5 years, 23 of 30 patients used a second splint after 19.9 ± 15.7 months, with a total of 2.7 ± 1.5 splints (Fig 2). Based on the last examination date of all subjects, the average PI was recorded as 0.7 ± 0.9.

In Table 2, a differentiation between PTSD patients with ST and a control group without ST is presented based on combat- and non-combat-associated reasons.

Treatment periods did not differ statistically significantly between combat and non-combat-induced PTSD patients nor between the different splint designs. Combat-induced PTSD patients were significantly older, had higher scores on the event scale, had a more significant extent of attrition, and the anamnestic timespan between the last traumatic event and the BL investigation was longer. The control group had a larger mouth opening at baseline and thus probably decided not to use the splint. Pain reduction in all groups was statistically significant during the first 6 weeks of hospitalised psychiatric and dental treatment. During further treatment in the ST group, the pain had decreased by the last examination/dental visit in the combat-induced PTSD group to 1.0 ± 1.3 on the VAS vs 0.9 ± 1.1 on the VAS in the non-combat-induced group.

Based on the last examination date of all subjects, the average PI on the VAS was recorded as 0.7 ± 0.9.

ST was performed on 113 patients with a modified acrylic interceptor and on 18 patients with a Michigan splint. No difference in pain reduction and mouth opening enlargement were recorded between the different splint designs after 6 weeks, and at the last appointment. After 41.1 ± 25.2 months. patients used 2.6 ± 1.1 Michigan splints or after 31.9 ± 30.4 months 2.0 ± 1.3 modified acrylic interceptor splints (Fig 2).

The data presented in this study are available on request from the corresponding author.

## DISCUSSION

A relationship between attritions and reduced mouth opening associated with painful TMJ and combat-related PTSD has been demonstrated.^2^^,^^6^ The association between symptoms of PTSD and signs of TMD in the German general population is well known.^21^ Especially in a period of international migration and foreign military assignments, it is important to increase knowledge/awareness of this in the field of dentistry.^19^

In a current systematic review with meta-analysis on post-traumatic stress and the prevalence of TMD in war veterans,^30^ 40 studies were identified based on adjunctive dental therapy. Those authors chose only four studies to obtain their results. The overall effect revealed that subjects exposed to war and diagnosed with PTSD had a higher prevalence of TMD signs (pain at muscle palpation) than controls (RR 2.21; 95 % CI:1.13–4.34), showing an association between war-related PTSD and TMD.^30^

Knibbe et al^22^ explored the association between types of traumatic events (war and combat-related vs non-combat-related PTSD) and the presence of painful TMD, awake (AB) or sleep bruxism (SB). Although exposure to these traumatic events was statistically significantly associated with painful TMD, AB, and SB after adjusting for confounders, the odds ratios were small and may still be a coincidence.^22^ However, research into this topic is limited and inconclusive.^7^^,^^8^^,^^15^^,^^17^

Treatment options for people experiencing painful TMD and bruxism include splints.^35^ They are provided to patients to help ease pain in the mouth, face, or jaw. They are also used to manage the symptoms of temporomandibular disorders, such as frequent headaches/migraines, clicking jaws, restricted mouth opening, or tooth wear from grinding the teeth (bruxism). There are many types of splints. Recent systematic reviews did not find sufficient evidence to support splint use for TMD or bruxism.^16^^,^^34^^,^^39^ Follow-up periods for outcome data of splint therapy in orofacial dysfunction were divided into short-term (0–3 months), medium-term (3–6 months), and long-term (6–12 months) follow-ups.^39^^,^^40^

When comparing oral splints with control splints, there was only very low-quality evidence from three studies^12^^,^^37^^,^^52^ that oral splints reduced pain compared with controls for a period of 0–3 months. However, this was not supported by results from the other periods of 3–6 months^9^^,^^20^^,^^32^^,^^33^^,^^45^^,^^49^ and 6–12 months.^9^^,^^31^^,^^25^^,^^32^^,^^33^^,^^47^ Nine studies reported on adherence to TMJ treatment with splints.^9^^,^^11^^,^^12^^,^^32^^,^^33^^,^^37^^,^^46^^,^^47^^,^^49^ Splints can reduce further non-carious loss of crown length and restorations.^26^ The combination of massage and splint therapy reduced the activity of the masseter and temporal muscles, while no reduction in PI occurred after 4 weeks.^14^^,^^23^^,^^39^^,^^40^^,^^53^

Reduction of TMD pain by adjunctive splint therapy with or without self-administered massage in patients with PTSD is documented for treatment times of 6 months.^13^^,^^28^^,^^44^^,^^51^

The present study presents the results of 30 patients for over 5 years and 72 patients for over 2 years. These outcomes should encourage general practitioners and dentists to consider the role of PTSD and traumatic events in the diagnosis and treatment of TMD associated with pain.

### Limitations of the Study

First and foremost, the study included only soldiers. Soldiers undergo mandatory dental examinations by the military to determine their dental fitness; however, dental treatment is compulsory only for deployment. Soldiers are at higher risk for the development of PTSD after military deployment all over the world.^6^ Thus, PTSD prevention efforts are still needed. Although the sample size was small, 153 of 180 soldiers with bruxism referred by the Bundeswehr Central Hospital Koblenz, Department and Center for Mental Health and Psychiatry to the Department of Dentistry for adjunctive dental treatment in PTSD over a span of 5 years were recruited. The empathy of military dentists towards their patients’ stress reaction to treatment needs for military deployment has not yet been analysed. It is currently unclear whether identifying bruxism-related TMD at an early stage could prevent burnout or other mental disorders following military deployment.^6^^,^^41^

The primary limitation of this study is the lack of exclusion or correction of former TMJ trauma. Another important limitation is the fact that painful TMD and bruxism were assessed with brief self-assessment questionnaires; as such, the results must be interpreted with caution.

The findings of the present study may not apply to the entire population of patients with PTSD, as it involved patients with severe PTSD who – up to the time of referral to the PTSD clinic – had been treatment-resistant.

Generalisation is also hampered by the fact that the study was conducted among a convenience sample of patients presenting for treatment at a PTSD clinic for different aetiological reasons (war exposure and others) with reflex/relaxation splint therapies.

## CONCLUSIONS

Fewer dental appointments visits and high tobacco use characterise dental care of PTSD patients prior to inpatient psychiatric therapy. This leads to a shift in care efforts with increased complexity of dental and psychiatric treatment.

Taking into account the retrospective recording of the last traumatising event, the average time of five years until therapy does not seem to have any consequences in terms of attrition defects in soldiers with PTSD. A tendency toward a higher extent of attrition exists in male PTSD patients. Pain reduction and the number of splints required during therapy did not differ between genders.

The extent of attrition, as well as more frequently omitted dental preventive measures due to dental phobias developed in PTSD patients, may lead to considerable dental treatment needs. A differentiation between awake and sleep bruxism (associated with headache in the morning) in combination with painful masticatory muscles and TMJ upon palpation is important for adjunctive dental TMD therapy with massage, exercise, and splints for relaxation. Splint therapy is effective in the reduction of PI and mouth opening. The presented study shows that the therapeutic short-term results achieved using a splint remain valid in the long term.

In conclusion, painful TMD was found to be more prevalent among patients with severe PTSD, with the severity of painful TMD being associated with the severity of PTSD symptoms. The present results suggest that oral health professionals may need to enquire about traumatic life events and PTSD symptoms and, if applicable, include trauma-focused dental treatment with massage, exercise of the TMJ and muscles, as well as reflex or relaxation splint therapy in the long term.

The results clearly demonstrated that war exposure, directly or indirectly, increases the risk of developing TMJ dysfunction and TMD signs/symptoms. People having experienced war-related trauma should seek help from mental health professionals and make lifestyle changes to reduce the risk of developing chronic conditions.

## Clinical relevance:

Most patients will benefit in the long term from a splint and remain free of painful TMD symptoms, even if PTSD persists.
